# Association Between Household Food Insecurity and Low Birth Weight: A Population-Based Case-Control Study

**DOI:** 10.34172/jrhs.2024.165

**Published:** 2024-09-30

**Authors:** Zahra Amiri, Masoumeh Sadeghi, Amin Moradi, Maryam Paydar, Mehdi Norouzi, Ehsan Mosafarkhani

**Affiliations:** ^1^Department of Epidemiology, School of Health, Mashhad University of Medical Sciences, Mashhad, Iran; ^2^Department of Environmental Health Engineering, School of Public Health, Mashhad University of Medical Sciences, Mashhad, Iran; ^3^Management and Social Determinants of Health Research Center, Mashhad University of Medical Sciences, Mashhad, Iran

**Keywords:** Low birth weight, Pregnancy outcome, Case-control study, Food security

## Abstract

**Background:** Low birth weight (LBW) is a major public health issue associated with increased neonatal morbidity and mortality. This study aimed to examine the association between household food insecurity and LBW in Mashhad, Iran.

**Study Design:** A population-based case-control study.

**Methods:** This study involved 6294 mothers (3247 cases and 3247 controls) who visited healthcare centers affiliated with Mashhad University of Medical Sciences for term births between March 2019 and March 2022. Cases included women who delivered neonates weighing<2500 g, while controls delivered babies≥2500 g. Food security was measured using the validated Household Food Insecurity Access Scale. Logistic regression models examined the associations between food insecurity and LBW. Geographic information system techniques mapped LBW distribution in Mashhad.

**Results:** Household food insecurity was significantly associated with higher odds of LBW (adjusted odds ratio [AOR]=1.25, 95% confidence interval [CI]: 1.03, 1.53). Other risk factors included younger maternal age (AOR=1.03, 95% CI: 1.01, 1.04), lower maternal education (AOR=0.55, 95% CI: 0.43, 0.71), cesarean delivery (AOR=1.70, 95% CI: 1.40, 2.06), and exposure to secondhand smoke (AOR=1.68, 95% CI: 1.02, 2.75). Gestational diabetes demonstrated a protective effect (AOR=0.37, 95% CI: 0.15, 0.91). Geographic mapping revealed that regions with higher food insecurity had elevated LBW prevalence.

**Conclusion:** These findings underscore the importance of addressing food insecurity among pregnant women to reduce the risk of LBW and improve newborn outcomes.

## Background

 Low birth weight (LBW), defined as a newborn weighing less than 2500 g, is a significant factor in neonatal mortality, accounting for approximately half of all perinatal deaths and one-third of neonatal deaths.^1^ Infants with LBW are at a higher risk of dying within the first 40 days of life compared to those with normal birth weight.^2,3^ Various maternal factors contribute to the risk of LBW, including extreme maternal age under 16 or over 40, multiple pregnancies, delivery complications, chronic maternal conditions such as prenatal hypertension, infections such as malaria, and poor nutritional status.^4-7^ Additionally, environmental exposures, such as indoor air pollution and tobacco use, further increase the risk.^8,9^

 Studies conducted in different regions of Iran have reported varying prevalence rates of LBW (11.8% in Qom,^10^ 19.1% in Hamedan,^11^ 8.6% in Tehran,^12^ 7.7% in Babol,^13^ 6.9% in Guilan,^14^ and 4.49% in Mashhad^15^), indicating inconsistencies and ambiguities in the prevalence of LBW among infants in Iran. Food security (FI) plays a crucial role in determining a child’s body mass index (BMI) and overall health. Research has shown a strong association between food security and health outcomes in children, impacting their growth and nutritional status.^16,17^ Ensuring food security is fundamental for good health, as it guarantees access to sufficient and nutritious food for all community members. Economic, financial, and familial challenges significantly affect children’s nutritional status.^18^ According to the Food and Agriculture Organization, around 2 billion people do not have regular access to safe and nutritious food, including 8% of the population in North America and Europe.^19^ This issue is particularly detrimental to pregnant women, who are especially vulnerable.^20^

 In Iran, FI is primarily investigated by measuring the adequacy of energy and nutrients using food questionnaires or by estimating the poverty line from income and expenditure surveys.^21^ FI poses a threat to the health and survival of individuals within communities and can have both short- and long-term effects.^22^ Several factors influence the prevalence of FI, including population growth, industrialization of communities, migration from rural to urban areas, inadequate levels of education, wars and economic sanctions by governments, pandemics or endemic diseases, and weather changes.^23-25^ A systematic review and meta-analysis (1990-2022) reported that food insecurity among the healthy Iranian population was 55.9%, with the highest prevalence in the western regions at 64.8%.^26^ A cross-sectional study by Khosravi et al revealed that 64.7% of women attending healthcare centers affiliated with Mashhad University of Medical Sciences (MUMS) had food security, while 25.3% experienced mild food insecurity, 6.7% moderate, and 3.4% severe food insecurity.^27^ Effective prenatal care and nutritional counseling are crucial for reducing the risk of LBW and achieving the sustainable development goals for 2030, which aim to lower neonatal mortality.^28,29^ Given the high prevalence of food insecurity, this study aims to investigate the association between food insecurity and LBW in a population-based study conducted at MUMS.

## Methods

###  Study design and data source

 This study was a population-based case-control study. The data were obtained from the Sina Electronic Health Record (SinaEHR) system, a comprehensive electronic health information system developed and implemented by MUMS. The SinaEHR system supports primary care delivery across various regions of Iran, covering a population of over 5 million people. It captures standardized clinical data, including diagnoses coded using the International Classification of Diseases, version 10 (ICD-10), and integrates laboratory results through a laboratory information system to facilitate real-time clinical decision-making.

###  Study participants

 Cases were mothers who delivered term neonates weighing < 2500 g at birth. Controls were mothers who delivered term neonates weighing ≥ 2500 g. The inclusion criteria for both cases and controls were live-born singletons delivered at term ( ≥ 37 weeks gestation). On the other hand, the exclusion criteria included neonates diagnosed with major congenital or genetic anomalies, mothers with a documented history of drug or alcohol abuse during the index pregnancy, and mothers with anemia (hemoglobin < 11 g/dL) during the index pregnancy. Cases and controls were frequency matched by maternal age (within 5-year age groups) and neonate gender, with a 1:1 ratio of cases to controls within each matched stratum. Approximately 85% of cases were matched to controls on both maternal age and neonate gender.

###  Measurement tools

 Data on various factors, including food security, smoking status, hypertension, domestic violence exposure, and diabetes, were collected using the Household Food Insecurity Access Scale, developed by the United States Agency for International Development to address the multifaceted nature of food security.^30^ The Persian validated version of this scale was utilized in the SinaEHR database. In the validation process, conducted by Moosavian et al,^31^ Mohammadi et al confirmed the internal reliability and validity of this questionnaire, with a Cronbach’s α of 0.855.^32^ The scale comprises nine items assessing food insecurity over the past four weeks, with responses rated on a two-point scale (yes = 1 or no = 0). Each item is followed by a query about the frequency of occurrence on a three-point scale (‘Rarely’, ‘Sometimes’, or ‘Often’), scored from 1 to 3, respectively. The frequency query is skipped if the response to the main item is “No”. The nine items encompass three main domains, namely, anxiety and uncertainty about food supply, insufficient food quality, and insufficient food intake and its physical consequences. The total Household Food Insecurity Access Scale score ranges from 0 to 27, with scores of 0-1, 2-7, 8-14, and 15 and above indicating food security, mild food insecurity, moderate food insecurity, and severe food insecurity, respectively. The Hurt, Insult, Threaten, Scream (HITS) domestic violence screening tool was employed to assess experiences of domestic violence. HITS comprises four items rated on a five-point Likert-type scale, with scores ranging from 1 to 5. Total HITS scores range from 4 to 20, with scores of 10 or higher implying a risk of domestic violence, a tool validated for research among Shiraz families.

###  Sample size and sampling technique

 The case and control groups each comprised 3247 individuals, selected through a census method. For each case included in the study, one control was selected by simple randomization. Stratified random sampling was utilized for control selection, with the target population divided into 20 strata based on geographic regions within Mashhad (referred to as Centers 1, 2, 3, 4, 5, and fifteen additional healthcare centers located in various cities across Khorasan Razavi, including Bakharz, Bardaskan, Binaloud, Chenaran, Dargaz, Khaf, Fariman, Qochan, Kalat, Kashmar, Khalilabad, Golbahar, Roshtkhar, Sarakhs, and Taybad).

###  Operational definitions and measurements

*Low birth weight*: Defined as the first weight of a newborn being less than 2,500 g at birth.

*Household food security*: Categorized as either food secure or food insecure based on the validated survey tool and labeled as secure and insecure.

###  Statistical analysis

 Data were entered into Excel (version 7.2.0.1) and imported into Stata (version 11) for analysis. Frequencies and percentages were calculated to summarize categorical variables. The outcome variable was coded as 1 for cases (LBW) and 0 for controls. Univariate analysis using chi-square tests was conducted to compare the distribution of independent variables between cases and controls. Multivariate logistic regression models were used to identify factors independently associated with LBW, with adjusted odds ratios and 95% confidence intervals estimated. Statistical significance was considered at *P* < 0.05. Collinearity between variables in the final model was assessed using standard diagnostic tests. The internal validation of the final multivariable model was performed using 10-fold cross-validation and bootstrap resampling (1000 replicates) to assess optimism and adjust for overfitting. Discrimination was evaluated using the area under the receiver operating characteristic curve, and calibration was determined using calibration plots and the Hosmer-Lemeshow goodness-of-fit test.

## Results

###  Demographic characteristics

 A total of 6494 mothers (3,247 cases and 3,247 controls) participated in the study. The mean ± standard deviation (SD) age of mothers was 30.9 ± 6.9 years. Among cases, the mean age was 31.45 ± 7.16 years compared to 30.41 ± 6.71 years in controls (*P*< 0.001). The majority of mothers were educated at the diploma level or above (n = 5,341, 82.25%), including 2,715 (50.83%) cases and 2626 (49.17%) controls. Most mothers were housewives (76.17%), comprising 819 (53.46%) cases and 713 (46.54%) controls.

 The mean ± SD BMI was 25.74 ± 5.17 kg/m^2^ overall. A total of 452 mothers (6.96%) had preeclampsia during pregnancy, including 344 (76.11%) cases and 108 (23.89%) controls. Approximately half of the cases (51.96%) had normal weight gain during pregnancy compared to 48.04% of controls. Cesarean delivery was common, reported by 1881 mothers (53.59%), with 1881 (58.69%) cases and 1324 (41.31%) controls. Food insecurity was present in 2,234 mothers (34.4%), including 1169 (52.33%) cases and 1,065 (47.67%) controls (Table 1).

**Table 1:** Distribution of low birth weight by demographic characteristics

**Continuous variables**	**Case (n=3247)**		**Control (n=3247)**		***P*** ** value**
	**Mean**	**SD**	**Mean**	**SD**	
Age of mother (y)	31.45	7.16	30.41	6.71	0.001
BMI (kg/m^2^)	25.58	5.37	25.90	4.97	0.010
Gestational age (wk)	37.37	0.01	38.49	0.01	0.001
Categorical variables	**Number**	**Percent**	**Number**	**Percent**	***P *** **value**
Education level					
Elementary and less	621	19.13	532	16.38	0.004
Diploma and more	2626	80.87	2715	83.62	
Maternal job					
Household	2428	74.78	2534	78.04	0.002
Employee	819	25.22	713	21.96	
History of cigarette smoking					
No history	2917	95.14	2922	96.06	0.081
Yes history	149	4.86	120	3.94	
Inactive smoking					
No	2275	95.87	2370	97.13	0.010
Yes	98	4.13	70	2.87	
Preeclampsia					
No	2903	89.41	3139	96.67	0.001
Yes	344	10.59	108	3.33	
Type of delivery					
Natural birth	1078	36.43	1,698	56.19	0.001
Cesarean delivery	1881	63.57	1,324	43.81	
Food security					
Secure	2078	64.00	2182	67.20	0.007
Insecure	1169	36.00	1065	32.80	
Neonate’s gender					
Girl	1783	54.91	1565	48.20	0.001
Boy	1464	45.09	1682	51.80	
Weight gain result					
Normal	1743	57.09	1878	60.80	0.003
Abnormal	1310	42.91	1211	39.20	
Domestic violence					
No	3192	98.92	3214	99.29	0.111
Yes	35	1.08	23	0.71	
Chronic blood pressure					
No	3058	98.68	3085	99.42	0.003
Yes	41	1.32	18	0.58	
Gestational diabetes					
No	3062	98.74	3059	98.77	0.913
Yes	39	1.26	38	1.23	

*Note*. BMI: Body mass index; SD: Standard deviation.

###  Food insecurity and low birth weight

Table 2 presents the odds ratios obtained from the logistic regression analysis, which was conducted to assess the association between various factors and LBW. Variables that were significant in the univariate analysis were included in the multivariate regression analysis. At this stage, variables with a significance level of 0.2 or less were added to the final model. The results of this analysis indicated a significant relationship between maternal education, maternal BMI, type of delivery, food security, preeclampsia, exposure to secondhand smoke (inactive smoking), and gestational age with LBW in both the case and control groups (*P*< 0.05). The final model had an area under the receiver operating characteristic curve of 0.78, indicating good discrimination and calibration (Hosmer-Lemeshow < 0.005) for the final model. A Brier score of 0.1875 represents reasonable predictive accuracy, and Spiegel Halter’s Z-Statistic (-0.2854, *P*= 0.612) indicates good calibration.

**Table 2:** Univariate and multivariate analyses to assess factors associated with low birth weight

**Variables**	**Unadjusted OR (95% CI)**	***P*** ** value**	**Adjusted OR (95% CI)**	***P*** ** value**
Maternal age	1.02 (1.01, 1.02)	0.001	1.00 (0.99, 1.02)	0.264
Neonate’s gender				
Girl	Ref.		Ref.	
Boy	0.76 (0.69, 0.84)	0.001	0.65 (0.56, 0.76)	0.001
Maternal job				
Household	Ref.		Ref.	
Employee	1.20 (1.07, 1.35)	0.001	1.09 (0.91, 1.32)	0.327
Maternal education				
Elementary and less	Ref.		Ref.	
Diploma and more	0. 82 (0.72,.94)	0.004	0.64 (0.56, 0.83)	0.001
Type of delivery				
Natural birth	Ref.		Ref.	
Cesarean section	2.23 (2.01, 2.48)	0.001	1.55 (1.32, 1.83)	0.001
Gestational age	0.71 (0.69, 0.72)	0.001	0.70 (0.68, 0.72)	0.001
Maternal BMI	0.98 (0.97, 0.99)	0.01	0.93 (0.92, 0.95)	0.001
Food security				
Secure	Ref.		Ref.	
Insecure	1.15 (1.04, 1.27)	0.007	1.21 (1.03, 1.43)	0.017
History of smoking				
No	Ref.		Ref.	
Yes	1.24 (0.97, 1.59)	0.082	1.12 (0.75, 1.66)	0.569
Inactive smoking				
No	Ref.		Ref.	
Yes	1.45 (1.06, 1.99)	0.018	1.92 (1.27, 2.91)	0.002
Preeclampsia				
No	3.44 (2.75, 4.30)	0.001	2.14 (1.56, 2.91)	0.001
Yes	Ref.		Ref.	
Weight gain result				
Normal	Ref.		Ref.	
Abnormal	1.16 (1.05, 1.29)	0.003	1.13 (0.97, 1.32)	0.102
Chronic blood pressure				
No	Ref.		Ref.	
Yes	2.29 (1.31, 4.01)	0.003	1.60 (071, 3.61)	0.250

*Note*. OR: Odds ratio; CI: Confidence interval; BMI: Body mass index.

###  Geographic information systems

**Figure 1:**
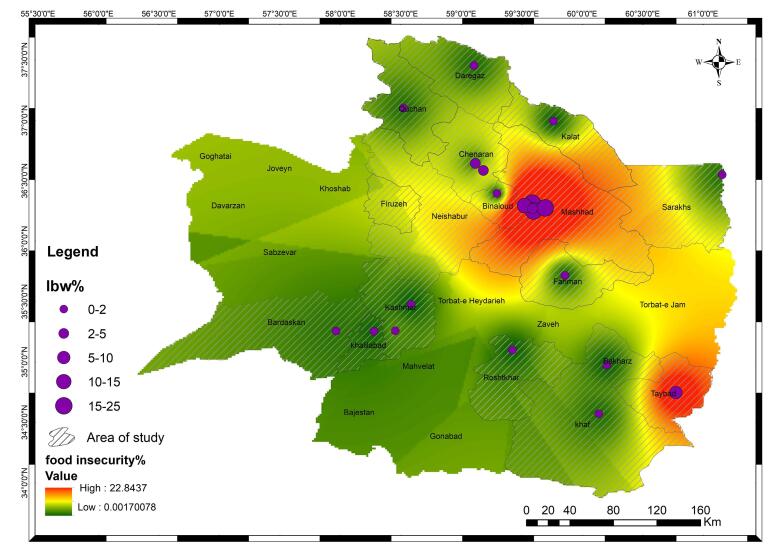
Distribution of low birth weight and food insecurity in Mashhad

 The analysis of geographic data visualized on a GIS map demonstrated that regions with a higher percentage of household food insecurity also exhibited higher rates of LBW. Among randomly selected health centers and cities in Razavi Khorasan Province, those with significant food insecurity—such as Taybad, Sarakhs, Kalat, Binaloud, and Neishabur—showed a corresponding pattern of elevated LBW prevalence. This geographic correlation highlights a clear overlap between community-level food insecurity and an increased risk of LBW (Figure 1).

## Discussion

 This study highlights the significant association between household food insecurity and an increased risk of LBW among term neonates. Even after adjusting for potential confounding factors, households experiencing food insecurity demonstrated a higher likelihood of delivering LBW infants compared to their food-secure counterparts. This finding aligns with the findings of research conducted in Addis Ababa, Ethiopia, rural Pakistan, and other parts of Ethiopia,^2,3,33^ as well as those of a cohort study from the United States^4^ and a case-control study in Tehran,^5^ all of which similarly linked food insecurity to an elevated risk of LBW. This association can be understood in light of the critical role that adequate nutrition plays during pregnancy, particularly in the second and third trimesters when fetal growth and development are most rapid. Food insecurity can deprive expectant mothers of essential nutrients necessary for optimal fetal growth, leading to intrauterine growth restriction and an increased risk of LBW.^34-37^ Additionally, the psychological stress associated with food insecurity may contribute to adverse pregnancy outcomes through physiological mechanisms such as increased inflammation, oxidative stress, and dysregulation of the hypothalamic-pituitary-adrenal axis.^20^

 The observed geographic correlation between areas with higher food insecurity and elevated LBW prevalence further underscores the potential impact of this socioeconomic determinant on birth outcomes. Communities struggling with food insecurity may face compounded challenges, including limited access to healthcare, suboptimal living conditions, and other socioeconomic disadvantages, which collectively contribute to adverse perinatal outcomes. Notably, the study also identified other significant risk factors associated with LBW, such as maternal smoking,^34^ preeclampsia, and chronic hypertension.^17-20^ These findings are consistent with existing literature, emphasizing the importance of comprehensive prenatal care and interventions targeting modifiable risk factors. Interestingly, gestational diabetes appeared to have a protective effect against LBW, a finding that warrants further investigation to explore the underlying mechanisms.

 The strengths of this study include its large sample size, population-based design, and the inclusion of a comprehensive set of potential confounding variables in the analysis. However, several limitations should be acknowledged:

- The retrospective nature of the study and the assessment of food security status based on a single time point may not fully capture the dynamic nature of household food insecurity during pregnancy.
- The reliance on retrospective data from a regional tertiary care center may limit the generalizability of the findings to a wider population.
- Unmeasured confounding factors, such as individual-level stress and inflammatory markers, could potentially influence the observed associations.
- The assessment of food security was based on a survey reflecting the preceding 12 months, which may fail to capture potential fluctuations during pregnancy.
- While the analysis adjusted for several confounding factors, the possibility of residual or unmeasured confounding factors remains (e.g., individual-level stress and inflammation markers).
- The case-control design establishes an association but does not prove causality between food access and LBW.
- Micronutrient deficiencies were not evaluated and may serve as an operational mechanism warranting further investigation.

**Highlights**

- The study identifies food insecurity as a significant risk factor for low birth weight (LBW), with a higher proportion of mothers in food-insecure households (52.33% cases vs. 47.67% controls) more likely to have LBW infants.
- GIS analysis confirms that regions with higher food insecurity levels correspondingly exhibit elevated rates of LBW, reinforcing the connection between food insecurity and LBW odds.
- These findings emphasize the need to address food insecurity at the community level to reduce the prevalence of LBW and improve maternal and child health outcomes.

## Conclusion

 In general, this study adds to the growing body of evidence linking household food insecurity to adverse birth outcomes, specifically LBW. The findings underscore the importance of addressing food insecurity as a critical public health issue with significant implications for maternal and child health. Targeted interventions, such as improving access to nutritious food for pregnant women and addressing underlying socioeconomic determinants, could play a pivotal role in reducing the burden of LBW and associated neonatal morbidity and mortality.^38,39^ Future prospective cohort studies are needed to further elucidate the causal pathways and potential mediating factors involved in this complex relationship.

## Acknowledgments

 This article is the result of a research project approved by MUMS, Iran, under the ethics code IR.MUMS.FHMPM.REC.1402.191 and grant number 4021102. We extend our gratitude to the Health Deputy of MUMS for their invaluable assistance in data collection.

## Authors’ Contribution

**Conceptualization:** Zahra Amiri and Ehsan Mosafarkhani.

**Data curation:** Amin Moradi and Mehdi Norouzi.

**Formal analysis:** Zahra Amiri.

**Funding acquisition:** Ehsan Mosafarkhani.

**Investigation:** Zahra Amiri, Masoumeh Sadeghi, and Ehsan Mosafarkhani.

**Methodology:** Zahra Amiri, Masoumeh Sadeghi, and Ehsan Mosafarkhani.

**Project administration:** Zahra Amiri.

**Resources:** Amin Moradi and Mehdi Norouzi.

**Software:** Zahra Amiri and Maryam Paydar.

**Supervision:** Ehsan Mosafarkhani and Masoumeh Sadeghi.

**Validation:** Ehsan Mosafarkhani and Masoumeh Sadeghi.

**Visualization:** Maryam Paydar.

**Writing–original draft:** Zahra Amiri.

**Writing–review & editing:** Zahra Amiri and Ehsan Mosafarkhani.

## Competing Interests

 None declared.

## Ethical Approval

 Approval for this study was obtained from the Ethics Committee of MUMS, with the approval code IR.MUMS.FHMPM.REC.1401.140.

## Funding

 This work was supported by MUMS under Grant 4021102.
